# Endogenously produced H_2_O_2_ is intimately involved in iron metabolism in *Streptococcus pneumoniae*

**DOI:** 10.1128/spectrum.03297-23

**Published:** 2023-12-01

**Authors:** Edroyal Womack, Babek Alibayov, Jorge E. Vidal, Zehava Eichenbaum

**Affiliations:** 1 Department of Biology, Georgia State University, Atlanta, Georgia, USA; 2 Department of Cell and Molecular Biology, University of Mississippi Medical Center, Jackson, Mississippi, USA; 3 School of Medicine, University of Mississippi Medical Center, Jackson, Mississippi, USA; Emory University School of Medicine, Atlanta, Georgia, USA

**Keywords:** *Streptococcus pneumoniae*, iron acquisition, heme degradation, hydrogen peroxide, bacteria physiology, iron metabolism, hemoglobin

## Abstract

In the presence of molecular oxygen, the human pathogen *Streptococcus pneumoniae* produces and secretes large amounts of hydrogen peroxide (H_2_O_2_), which can readily interact with free and heme-bound iron. Here, we investigated the role of the endogenously produced H_2_O_2_ in iron acquisition. The data revealed that *S. pneumoniae* uses H_2_O_2_ to liberate iron from met-hemoglobin (Hb-Fe^3+^) extracellularly, allowing the bacterium to import and grow on free iron even when cultivated on met-hemoglobin as the only iron source. The loss of H_2_O_2_ production leads to a dramatic pneumococcal intake of heme and is associated with a robust upregulation of most iron uptake machinery (indicating an iron starvation signal). These and other data reveal a close and previously unexplored relation between H_2_O_2_ production and iron metabolism in *S. pneumoniae*. The data also show that, in addition to extracellular degradation, pneumococci are armed with H_2_O_2_-independent mechanisms for intracellular heme catabolism.

**IMPORTANCE:**

Heme degradation provides pathogens with growth essential iron, leveraging on the host heme reservoir. Bacteria typically import and degrade heme enzymatically, and here, we demonstrated a significant deviation from this dogma. We found that *Streptococcus pneumoniae* liberates iron from met-hemoglobin extracellularly, in a hydrogen peroxide (H_2_O_2_)- and cell-dependent manner; this activity serves as a major iron acquisition mechanism for *S. pneumoniae*. Inhabiting oxygen-rich environments is a major part of pneumococcal biology, and hence, H_2_O_2_-mediated heme degradation likely supplies iron during infection. Moreover, H_2_O_2_ reaction with ferrous hemoglobin but not with met-hemoglobin is known to result in heme breakdown. Therefore, the ability of pneumococci to degrade heme from met-hemoglobin is a new paradigm. Lastly, this study will inform other research as it demonstrates that extracellular degradation must be considered in the interpretations of experiments in which H_2_O_2_-producing bacteria are given heme or hemoproteins as an iron source.

## INTRODUCTION

*Streptococcus pneumoniae* (pneumococcus) is a catalase-negative facultative anaerobe that continues to cause severe illness and mortality despite vaccine availability. Pneumococcus is carried asymptomatically in the nasopharynx of up to 65% of the population. In some carriers, the bacterium translocates through mucosal, epithelial, and endothelial barriers and proliferates at otherwise sterile sites such as the middle ear, lungs, blood, meninges, and the endocardium. Each year, pneumococcus accounts for ~10.6 million invasive infections, causing ~2 million deaths, of which children and the elderly are most impacted (1 – 3). With the emergence of SARS-CoV-2, bacterial co-infections were prevalent in COVID-19 patients with *S. pneumoniae* the most common infection partner (4, 5).

The total iron pool in human adults is around 3–4 g of which about 75% is in the form of heme mostly bound to hemoglobin (6). To avoid toxicity and overcome solubility problems, the host sequesters virtually all the iron in the extracellular and intracellular milieus using specialized proteins that transport, bind, or store iron or heme. During infection, iron availability is further decreased due to a coordinated host response named nutritional immunity (7, 8). Hence, invading bacteria that require iron, such as *S. pneumoniae*, rely on dedicated mechanisms to capture the metal from host proteins. We recently showed that heme, hemoglobin, and several other host heme sources restore pneumococcal growth in an iron-depleted medium. Notably, hemoglobin promotes growth *in vitro* to a greater capacity than other iron or heme sources and supported growth in concentrations that were toxic with equivalent amounts of free heme (9). In addition to facilitating vigorous planktonic proliferations, hemoglobin induces early and robust pneumococcal biofilms *in vitro* and drives global transcriptome changes enabling the pathogen adaptation to the host mucosal environment (10). These and other observations established hemoglobin as a principal nutrient and host signal for pneumococci (9).

Gram-positive pathogens often employ protein-relay machinery consisting of surface proteins that capture the heme from the host and deliver it across the thick cell wall and eventually to the substrate-binding component of a membrane-bound ABC transporter (11). The molecular mechanisms for heme or iron acquisition are not fully explained in *S. pneumoniae*. Surface receptors such as those that shuttle heme across the cell wall in other Gram-positive bacteria have not been described in this human pathogen. Several ABC transporters involved in iron uptake were identified, but the *in vivo* ligand of some transporters remained undetermined. The two pneumococcal ABC systems proposed to import ferric iron are the pit1ADBC (spd_0223–0227) and pit2ABC (spd_1607–1609) transporters (12, 13). In the D39 strain, the substrate-binding component, pitA1 is truncated, but the permeases and ATP-binding protein are intact. *S. pneumoniae* also encodes the *pia* transporter of which PiaA, the ligand-binding protein, was crystalized with ferrochrome (14). Previous studies also demonstrated that the Pia proteins support pneumococcal growth on heme iron (15). Similarly, the PiuBCDA transporter promotes streptococcal binding to heme and hemoglobin (16), but the substrate-binding component, PiuA, binds *in vitro* norepinephrine and enterobactin in higher affinity than heme (17). A recent study implicated an additional pneumococcal ABC transporter, *spd_0088–0090*, in heme uptake (18). Due to the redundancy of iron uptake mechanisms, genetic knockouts in multiple transport systems are required before attenuated pneumococcal growth is observed (13).

Several studies reported the presence of free hemoglobin in the mucosa of the upper respiratory tract due to bleeding and erythrocyte lysis (19). In intact erythrocytes, hemoglobin iron is found in its reduced form (ferrous, Fe^+2^) bound to oxygen (oxyhemoglobin) or carbon dioxide. Upon erythrocytes lysis, the iron in oxyhemoglobin is autoxidized to ferric (Hb-Fe^+3^) forming met-hemoglobin, which cannot bind oxygen (20). Oxyhemoglobin interactions with hydrogen peroxide (H_2_O_2_) lead to heme degradation; these reactions start with the formation of ferryl hemoglobin (Hb-Fe^+4^=O), which in turn mediates one-electron oxidation of H_2_O_2_ creating a superoxide radical in the heme-binding pocket. Subsequent reactions with the porphyrin ring degrade the heme and release free iron and two fluorescent products (20, 21). On the other hand, met-hemoglobin interactions with H_2_O_2_ do not lead to heme degradation but catalytically consume the H_2_O_2_ (20). In these catalase-like reactions, one H_2_O_2_ molecule serves as a two-electron acceptor leading to the formation of oxo-ferryl complex (Fe^+4^=O) and transient apoprotein radicals (e.g., *Hb-Fe^+4^=O) while a second H_2_O_2_ molecule serves as a two-electron donor that reduces the ferryl hemoglobin back to met-hemoglobin while producing molecular oxygen (O_2_) (20, 22, 23).

In the human host, pneumococcus is exposed to oxygen levels that vary from 20% to 5% in the upper to lower respiratory tracts to virtually no free oxygen in the blood (24, 25). In the presence of oxygen, *S. pneumoniae* produces H_2_O_2_ as a byproduct of the enzymatic reactions of the pyruvate oxidase (*spxB*) and lactate oxidase (*lctO*) (26, 27). The SpxB and LctO enzymes are responsible for ~80% and ~20% of the pneumococcal H_2_O_2_ production, respectively (28). SpxB activity augments the pneumococcal release of Ply, a pore-forming toxin, which promotes erythrocyte lysis (29), likely increasing the availability of free hemoglobin in the immediate bacterial environment. Our group demonstrated that *spxB* is highly expressed during growth with hemoglobin (9). We showed that endogenously produced H_2_O_2_ catalyzes the oxidation of oxyhemoglobin to met-hemoglobin and provided spectroscopic data supporting the H_2_O_2_-mediated degradation of the heme in oxyhemoglobin from rupturing erythrocytes (30). Still, the impact of H_2_O_2_ on met-hemoglobin, the dominant form of hemoglobin in the extracellular compartment, and on pneumococcal iron acquisition overall was not described. In this study, we aim at these knowledge gaps and show that *S. pneumoniae* uses H_2_O_2_ to acquire iron from met-hemoglobin and that H_2_O_2_ production is closely linked to iron and heme metabolism in this key human pathogen.

## RESULTS

### The interactions between H_2_O_2_ and met-hemoglobin promote their removal from the medium during pneumococcal growth

**Fig 1:**
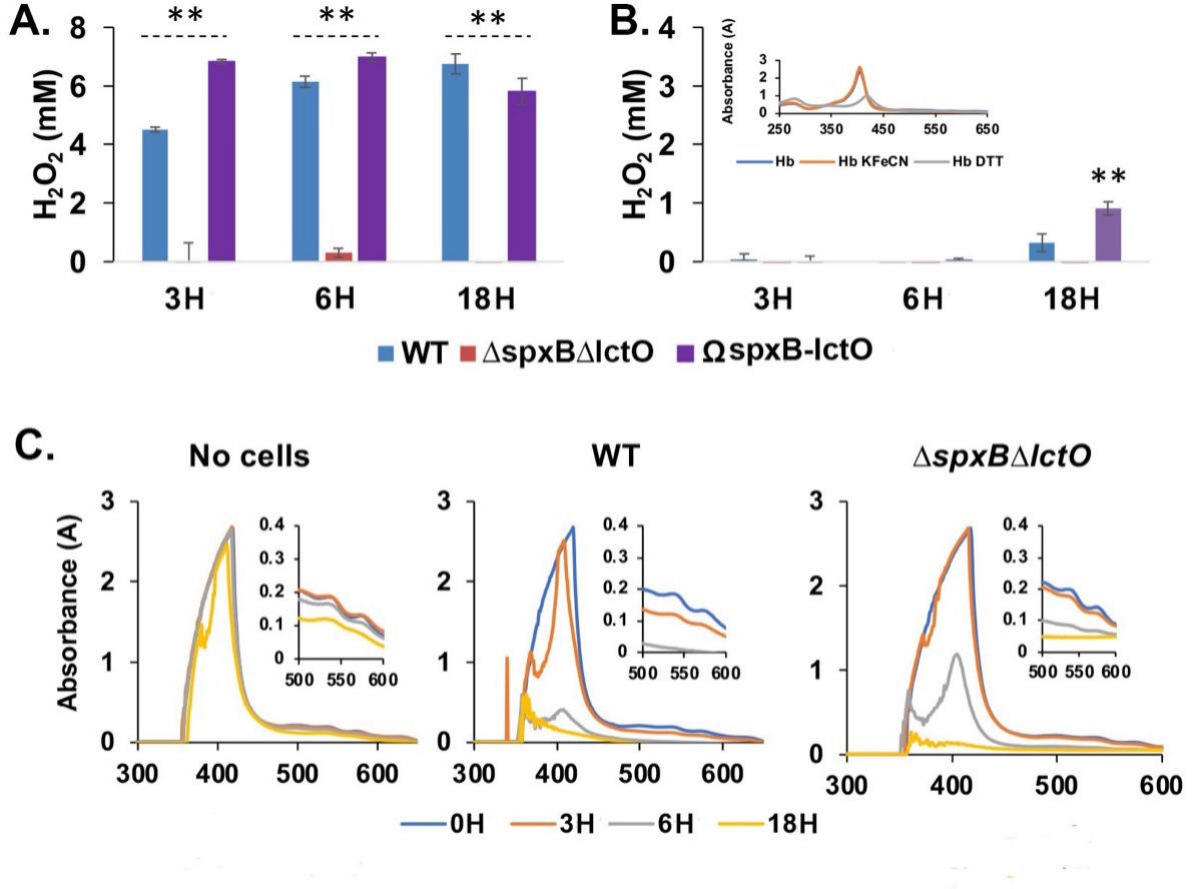
H_2_O_2_ and met-hemoglobin consumption during pneumococcal growth on met-hemoglobin iron. Shown are H_2_O_2_ concentrations in the culture supernatant of D39 WT, *∆spxB∆lctO* mutant, and complemented (Ω*spxB-lctO*) strains grown in THYB, (**A**) or (**B**) in THYB supplemented with 20-µM met-hemoglobin (Hb). The insert contains the absorbance spectra of 10-µM hemoglobin stock solutions in PBS (Hb), PBS with 30-µM potassium ferricyanide (Hb FeCN) or 10-mM dithiothreitol (Hb DTT). (**C**) The absorbance spectra of un-inoculated THYB containing 10-µM Hb, THYB 10-µM Hb inoculated with the D39 WT or the *∆spxB∆lctO* mutant incubated at 37°C for up to 18 hours (**H**), spent THYB medium used as blank. Cells were removed by centrifugation prior to absorbance reading. The data were derived from experiments done in duplicates and repeated twice and analyzed by analysis of variance, where * indicates *P*  ≤  0.05 and ** indicates *P* ≤ 0.01.

Since free oxyhemoglobin is rapidly oxidized by H_2_O_2_ (31, 32), we rationalized that *S. pneumoniae* encounters mostly met-hemoglobin in the respiratory mucosa. To begin investigating the impact of H_2_O_2_ production on pneumococcal interactions with met-hemoglobin and iron uptake, we constructed a double ∆*spxB*∆*lctO* mutant and a complemented strain (expressing *sp*x*B* and *lctO* genes from a heterologous location in the chromosome) in the background of the D39 strain. We monitored the H_2_O_2_ levels in the culture media during growth and confirmed that the *∆spxB∆lctO* mutant did not produce H_2_O_2_ at detectable levels, while we found 5–7 mM of H_2_O_2_ in the culture supernatant of wild-type (WT) and the complemented strains (Fig. 1A).

We next examined the H_2_O_2_ amount in pneumococcal cultures growing in the presence of met-hemoglobin (Fig. 1B). We solubilized lyophilized hemoglobin (Sigma) in phosphate buffer saline (PBS) and determined the absorbance spectrum following incubation with oxidative (potassium ferricyanide) or reducing (DTT) agents. The Soret peak of the hemoglobin incubated with or without potassium ferricyanide overlapped. In the presence of DTT, however, the absorbance maximum shifted right from 408 to 418 nm, indicating iron reduction (Fig. 1B, insert). These spectral characteristics indicate that the hemoglobin in our stock solution is in the oxidized (met-hemoglobin) state. Analysis of the H_2_O_2_ levels in the culture medium revealed that when the WT and complemented strains were grown in Todd-Hewitt broth (THYB) supplemented with 20-µM met-hemoglobin, H_2_O_2_ was not detectable at the 3- and 6-hour time points (Fig. 1B). Hence, the H_2_O_2_ produced by pneumococci is removed, likely due to reactions with the medium met-hemoglobin. Low amounts of H_2_O_2_ (10%–25% compared to THYB) were detected after 18 hours suggesting met-hemoglobin depletion during incubation allows for some accumulation of H_2_O_2_ at the later time point.

While the hemoglobin remains soluble in un-inoculated THYB, visual inspection suggested that some of the met-hemoglobin is falling out of solution, with more precipitation occurring in the WT cultures compared to the *∆spxB∆lctO* mutant. Hemoglobin precipitation was confirmed by centrifugation and washes of the cell pellet. We used spectroscopic measurements to follow the levels of the hemoglobin that remained in solution during growth. The hemoglobin displayed a somewhat broad Soret peak, suggesting that some of the heme iron in the hemoglobin was reduced by the medium. Nevertheless, we did not see distinct changes in the Soret maximum during incubation in regular THYB even after 18 hours of incubation. On the other hand, in a medium that was inoculated with the WT *S. pneumoniae*, the hemoglobin spectrum maximum was decreased and left shifted in the first 3 hours forming the narrow peak at 408 nm, which is typical of oxidized hemoglobin (met-hemoglobin). The Soret peak decreased dramatically within the next 3 hours of incubation and eventually was below the detection level by the 18-hour time point (Fig. 1C). In the ∆*spxB*∆*lctO* culture, the changes in the Soret happened at a much lower rate; no changes in absorption were observed in the first 3 hours of incubation. While the Soret peak decreased during longer incubation, it was significantly higher compared to the Soret maximum with the WT strain at the 6-hour time point. Altogether, the data suggest that H_2_O_2_ produced by *S. pneumoniae* interacts with the met-hemoglobin in the medium via reactions that remove both H_2_O_2_ and the protein. Still, mechanisms that are independent of H_2_O_2_ also contribute to the reduction in the levels of soluble met-hemoglobin in the pneumococcal growth medium.

### H_2_O_2_ reaction mediates iron release from met-hemoglobin in a cell-dependent manner

**Fig 2:**
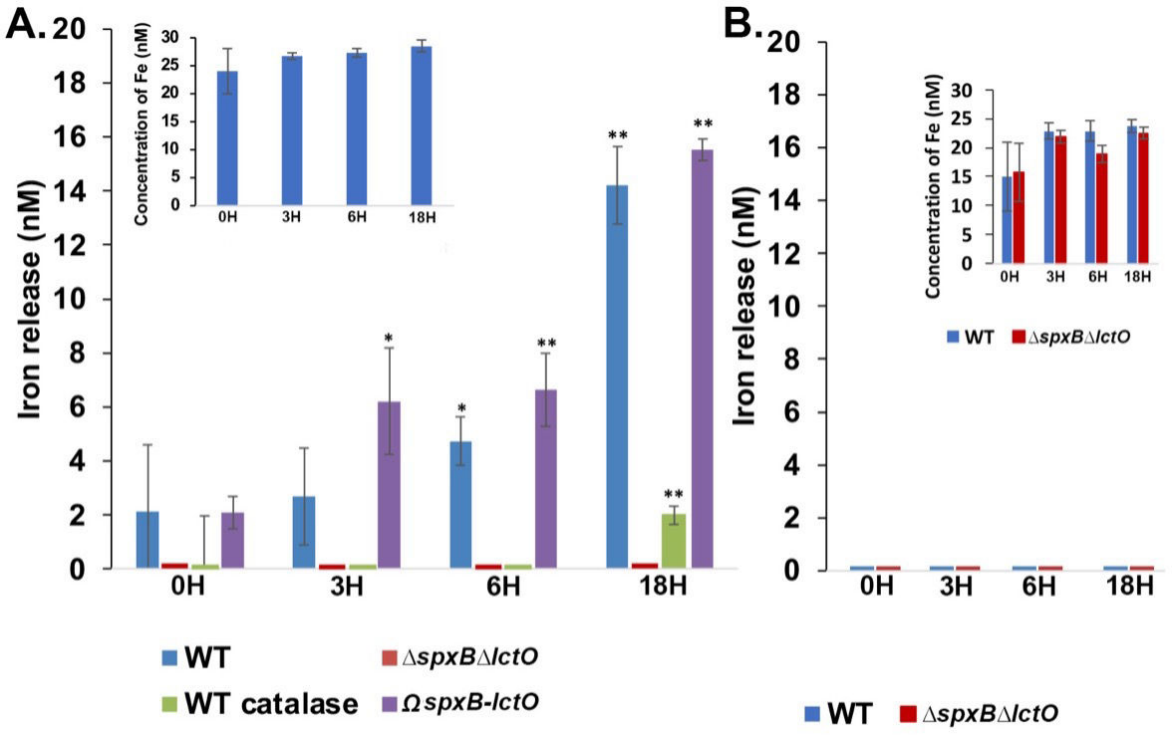
H_2_O_2_ produced by *S. pneumoniae* acts to liberate iron from met-hemoglobin in a cell-dependent manner. (**A**) Un-inoculated THYB supplemented with 20-µM met-hemoglobin or medium inoculated with isogenic D39 WT, *∆spxB∆lctO*, and the complemented strain (Ω*spxB-lctO*) were incubated at 37°C. WT pneumococci were used to inoculate also THYB with 20-µM met-hemoglobin containing 200-U/µL catalase (WT catalase). Free iron levels in media samples collected at different time points were determined after removing the cells by centrifugation and the hemoglobin by filtration. Insert shows iron concentrations in un-inoculated THYB incubated with 20-µM met-hemoglobin. Net iron released into the culture media was calculated by subtracting the iron concentration found in un-inoculated medium at the same time point. (**B**) A 20-µM met-hemoglobin was added to supernatant samples collected from overnight cultures of D39 WT or *∆spxB∆lctO* strains, and the samples were allowed to incubate at 37°C. The iron concentration in each sample was determined after the hemoglobin was removed by filtration (as in A). Insert shows the concentration of free iron in the reactions. Subtracting the iron concentration found in un-inoculated THYB with 20-µM met-hemoglobin (insert in A) from those found in spent media incubated with hemoglobin revealed no net iron release. Data were derived from experiments done in duplicates and repeated twice and analyzed by student *t*-test, where * indicates *P*  ≤  0.05 and ** indicates *P* ≤ 0.01.

We next tested if the interactions between the endogenously produced H_2_O_2_ and met-hemoglobin release iron into the culture supernatant. THYB supplemented with 20-µM met-hemoglobin was allowed to incubate at 37°C. The hemoglobin was then removed by filtration, and the concentration of free iron in the medium was determined at different time points using the chromophore ferrozine (33). The addition of met-hemoglobin to THYB led to an immediate release of small amounts of iron (~20 nM), but no significant change in iron levels was observed from that point during incubation (insert in Fig. 2A). Hence, it seems possible that a minute fraction of the hemoglobin solution contains free iron. Still, the hemoglobin was generally stable in THYB and did not spontaneously release iron during incubation in THYB at 37°C. Notably, when THYB supplemented with 20-µM met-hemoglobin was inoculated with the WT D39 cells, the free iron concentration continued to rise above the baseline observed with an un-inoculated medium. The addition of catalase prevented this rise in iron level in the early time points, and minuscule amounts of additional iron were detected after 18 hours (Fig. 2A). Moreover, we did not observe iron release from met-hemoglobin into the medium in the ∆*spxB*∆*lctO* culture, while the complemented strain released iron in higher amounts compared with the WT strain (Fig. 2A). Hence, H_2_O_2_ produced by the growing pneumococci acted to release iron from met-hemoglobin into the extracellular environment.

To examine if H_2_O_2_ was sufficient to cause iron release in the pneumococcal culture, we added met-hemoglobin into the supernatant samples collected from overnight cultures of the WT (which contains ~7-mM H_2_O_2_, Fig. 1A). Spent medium collected from *∆spxB∆lctO* culture served as a negative control. Interestingly, we did not see an increase in free iron during incubation of met-hemoglobin in either spent media (Fig. 2B). Hence, endogenously produced H_2_O_2_ aids iron release, but it is not exclusively sufficient to release iron from the externally added met-hemoglobin as this process also requires the bacterial cells.

### *S. pneumoniae* grown on met-hemoglobin as an iron source imports mostly free iron

**Fig 3:**
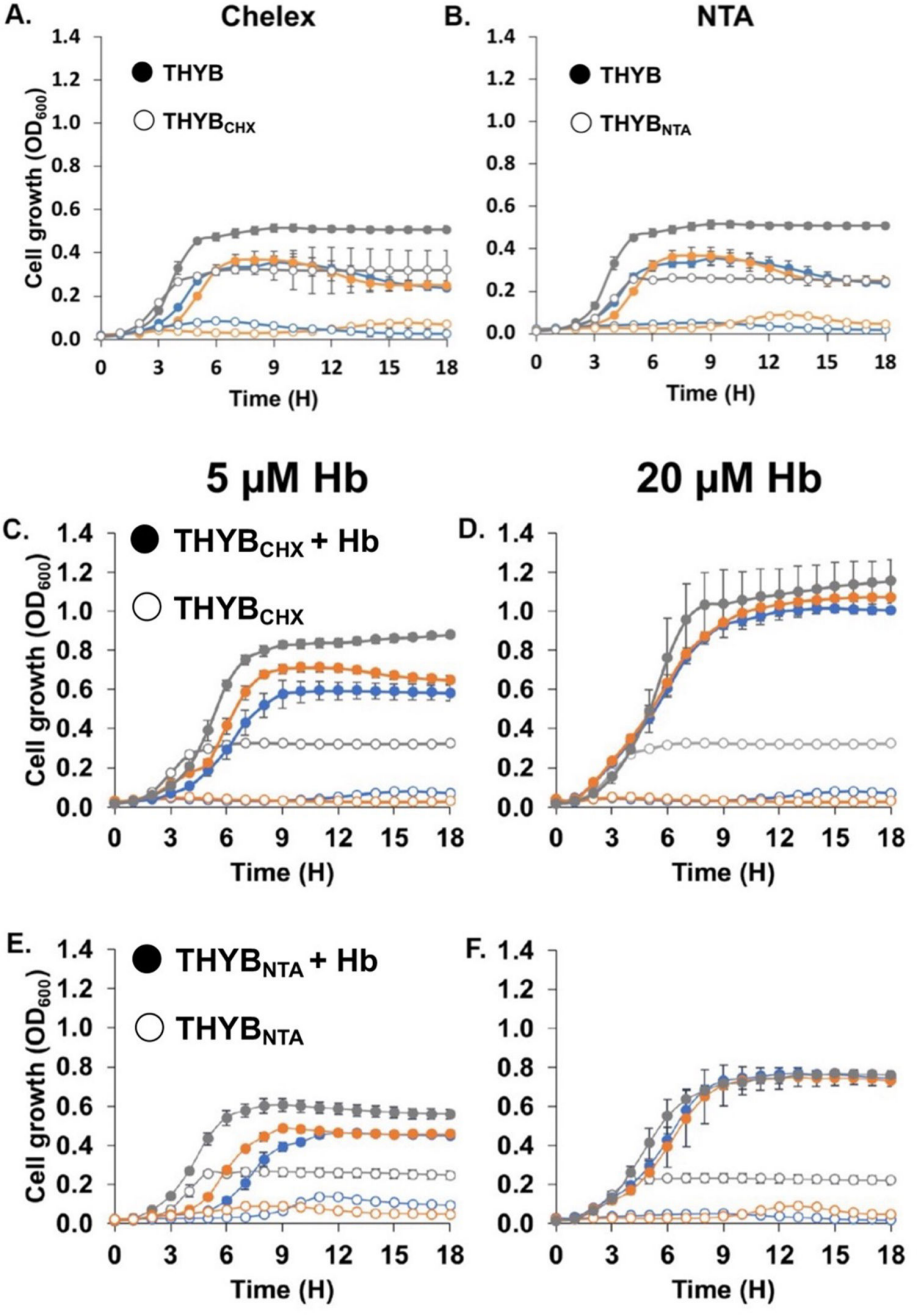
*S. pneumoniae* growth using met-hemoglobin as the sources of iron. Cells of the isogenic strains D39 WT (blue), *∆piuBCDA* (orange), and *∆spxB∆lctO* (gray) were used to inoculate fresh media in microtiter plates at OD_600_ 0.05. The cultures were incubated at 37°C, and the optical density was recorded. *S. pneumoniae* was cultivated in regular THYB or iron-depleted THYB supplemented with met-hemoglobin (Hb) (full symbols) or in iron-depleted THYB (empty symbols). Iron was depleted from the medium by treating it with Chelex-100 (THYB_CHX_) or by the addition of 3 mM of the iron chelator, NTA (THYB_NTA_). Shown is pneumococcal growth in THYB and THYB_CHX_ (**A**), THYB and THYB_NTA_ (**B**), and THYB_CHX_ and THYB_CHX_ with (**C**) 5- or (**D**) 20-µM met-Hb and THYB_NTA_ and THYB_NTA_ with (**E**) 5- or (**F**) 20-µM met-Hb. Experiments were done in triplicates and repeated at least two more times. Each curve shown is derived from an average of three bioreplicates from a representative experiment.

To better understand how H_2_O_2_ production impacts *S. pneumoniae* use of met-hemoglobin as an iron source, we aimed to determine the heme and iron content in cells growing on met-hemoglobin iron. For this, we explored two separate growth assays in which we depleted the free iron from the medium by either using THYB pretreated with Chelex-100 resin (THYB_CHX_, Fig. 3A) or in THYB containing the iron chelator, nitrilotriacetic acid (NTA) (THYB_NTA_, Fig. 3B). The main difference between these two growth assays is that with THYB_CHX_, the iron is removed ahead of inoculation from the medium. Hence, when met-hemoglobin is added (Fig. 3C and D), the bacteria can use both the heme and free iron (if it is freed into the supernatant during growth). In THYB_NTA_, however, the only source of iron that is available for pneumococcus is the met-hemoglobin (Fig. 3E and F), since NTA is in excess and will chelate any iron released into the medium.

Chelating the iron in THYB either by Chelex or by NTA impaired pneumococcal growth (Fig. 3, empty symbols). The addition of 5- and 20-µM met-hemoglobin to the media restored the growth of both the WT and the *∆spxB∆lctO* strains in a dose-dependent manner (Fig. 3C–F). Hence, growth arrest in THYB_CHX_ or THYB_NTA_ resulted from iron depletion. Moreover, *∆spxB∆lctO* growth in an iron-depleted medium supplemented with met-hemoglobin demonstrates that H_2_O_2_ production is not essential for pneumococcus to use met-hemoglobin as an iron source and the pathogen has additional means to liberate iron from heme.

**Fig 4:**
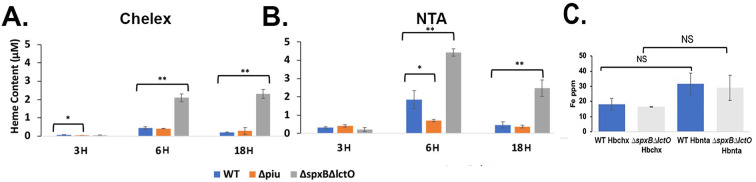
Heme and iron cellular content in pneumococci grown using met-hemoglobin as the source of iron. Cells of the isogenic strains D39 WT (blue), *∆piuBCDA* (orange), and *∆spxB∆lctO* (gray) were allowed to grow in THYB_CHX_ or THYB_NTA_ supplemented with 20-µM met-hemoglobin (as described in Fig. 3). Cells harvested at different time points were washed, and the heme contants were determined by the acidified chloroform method. Shown are the cellular heme contents in pneumococci grown in met-hemoglobin supplemented THYB_CHX_ (**A**) or THYB_NTA_ (**B**). Intracellular iron content measured by ICP-MS in cell samples collected at the 6-hour time point is shown in **C**. The data (normalized to optical density) are derived from experiments done in duplicates and repeated twice. The Student *t*-test was used to determine statistical significance, where * indicates *P*  ≤  0.05 and ** indicates *P* ≤ 0.01; NS indicates not significant.

We next measured the heme content in cells collected at different time points during cultivation in THYB_CHX_ or THYB_NTA_ supplemented with 20-µM met-hemoglobin (Fig. 4A and B). Respectively, the tested strains accumulated heme with the heme levels peaking at the logarithmic phase of growth (6 hours) and then reduced by the 18-hour time point. The WT strain contained more heme when grown in THYB_NTA_ compared with THYB_CHX_, exhibiting four times more heme at the late logarithmic phase of growth (Fig. 4B and A, respectively). We also tested heme accumulation by a knockout of the *piuBCDA* system, previously implicated in heme import. The *∆piuBCDA* strain imported significantly less heme than the WT, when grown in THYB_NTA_ (Fig. 4B), confirming, for the first time, that the Piu proteins contribute to heme accumulation *in vivo*. Interestingly, cellular heme levels in the WT and *∆piuBCDA* were comparable during growth in THYB_CHX_ (Fig. 4A).

The most noticeable difference in heme content was exhibited by the *∆spxB∆lctO* mutant, which accumulated much more heme than the other strains (fivefold more than the WT in THYB_CHX_ and twofold more in THYB_NTA_, Fig. 4A and B). Moreover, the cellular levels of heme in this strain remained high even after 18 hours of incubation. Like the WT and *∆piuBCDA*, the ∆*spx*∆*lctO* strain also imported more heme when grown in THYB_NTA_ compared with THYB_CHX,_

Despite the big difference in heme content between the WT and the *∆spxB∆lctO* strains when grown on met-hemoglobin iron in either THYB_CHX_ or THYB_NTA_, inductively coupled plasma mass spectrometry (ICP-MS) analysis (done at the 6-hour time point) did not reveal meaningful difference in cellular iron content between these two strains (Fig. 4C). We observed an upward trend in total iron in cell samples collected from THYB_NTA_ compared to those collected from THYB_CHX_, but the difference was not statistically significant. Therefore, the WT strain imported mostly free iron when grown on met-hemoglobin iron. In the absence of extracellular heme degradation, the *∆spxB∆lctO* imported heme to fulfill its iron needs. Importantly, the two strains balance iron and heme uptake and keep total cellular iron levels at a steady state regardless of the iron source available to them.

**Fig 5:**
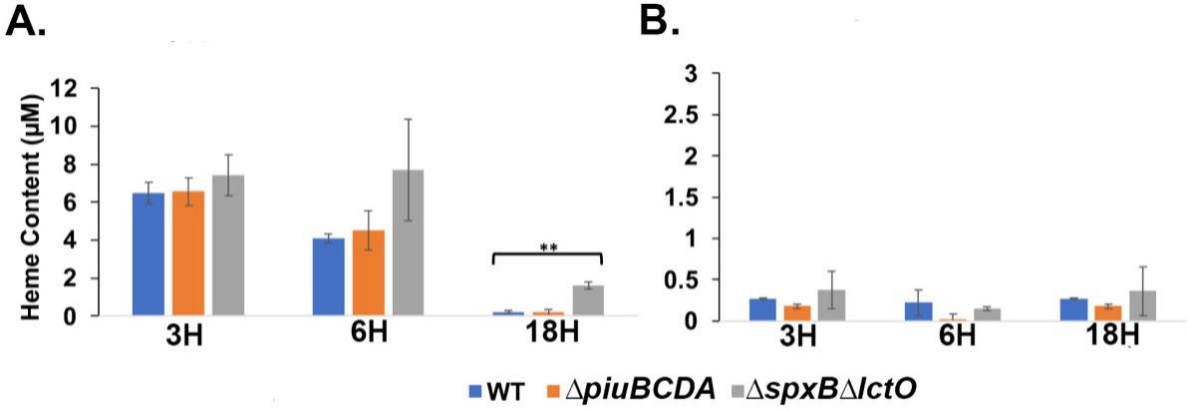
The heme in culture medium containing met-hemoglobin is spent faster by H_2_O_2_-producing *S. pneumoniae*. Cells of the isogenic strains D39 WT (blue), *∆piuBCDA* (orange), and *∆spxB∆lctO* (gray) were allowed to grow in THYB_NTA_ supplemented with 20-µM met-hemoglobin (as described in Fig. 3). Culture samples (2 mL) were collected along the growth, the cells were removed by centrifugation, and total heme content was determined by the acidified chloroform method (**A**), or the hemoglobin was removed by filtration prior to determination of the free heme concentration (**B**). The data were derived from experiments done in duplicates and repeated twice. The Student *t*-test was used to determine statistical significance, where * indicates *P*  ≤  0.05 and ** indicates *P* ≤ 0.01.

We also evaluated the total heme concentration (i.e., bound to met-hemoglobin and free) in the medium during growth of the WT, *∆piuBCDA*, or *∆spxB∆lctO* strains in THYB_NTA_ supplemented with met-hemoglobin (Fig. 5A). In these experiments, we removed the cells and then extracted the heme from the medium using acidified chloroform as described (34). In the cultures of the WT and *∆piuBCDA* strains, about 30% of the heme found in the medium after 3 hours of growth was spent by the 6-hour time point and was almost completely diminished after 18 hours of incubations. The heme was also removed during the growth of the *∆spxB∆lctO* cells but at a lower rate. The culture supernatant of this strain contained ~33% more heme than those of the WT and *∆piuBCDA* strains even after 18 hours (Fig. 5A). To determine the fraction of free heme (unlike heme that is bound to met-hemoglobin), we repeated the assay after removing the met-hemoglobin by filtration (Fig. 5B). We found only minute amounts (~4% of the total heme) of free heme in the medium of all cultures, indicating that most of the heme remaining in the supernatant at any time point is still bound to met-hemoglobin.

### Inactivation of H_2_O_2_ production drives overexpression of iron and iron-complex importers

**Fig 6:**
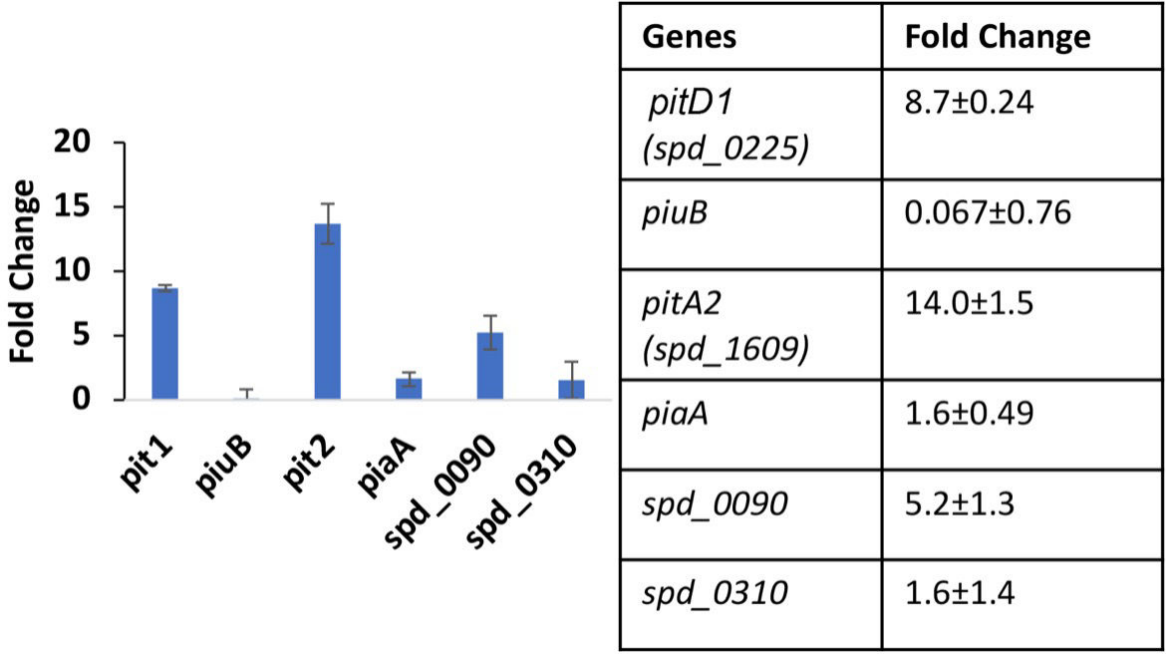
The *∆spxB∆lctO* mutant overexpress iron uptake genes compared with the WT strain. Shown are the fold changes in the expression of iron uptake and shuttling genes in the *∆spxB∆lctO* mutant compared to the isogenic WT strain as determined by qRT-PCR. RNA was prepared from cells grown in THYB up to the mid-log phase of growth. Data derived from two bioreplicates and processed in duplicates are shown in a bar graph and table format.

The growth of the *∆spxB∆lctO* mutant was significantly impaired in THYB_CHX_ and in THYB_NTA_ compared with regular THYB (Fig. 3A and B, gray symbols,). Still, the *∆spxB∆lctO* mutant exhibited limited growth in these iron-depleted media (gray empty symbols), where the growth of the WT strain was completely diminished (blue empty symbols), indicating that it is more resistant to iron depletion. To compare iron metabolism in these two strains, we compared the expression of iron-related genes during growth in regular THYB. RNA was prepared from cell samples collected at the mid-logarithmic phase, and gene expression was analyzed by reverse transcription PCR (RT-PCR). We observed strong activation of several iron and heme importers in the *∆spxB∆lctO strain* (Fig. 6). This includes the activation of *pitA2* (14-fold), *pitD1* (8.7-fold), *spd_0090* (5.2-fold), and *pia*A (1.6-fold). The expression of *spd_0310* (35), encoding a putative heme shuttling protein, was also induced 1.6-fold. Curiously, the *piuB* gene was strongly downregulated (0.067-fold) in the *∆spxB∆lctO* mutant. Hence, inactivation of the *spxB and lctO* has a significant impact on iron homeostasis in *S. pneumoniae*.

### Pneumococci growing on met-hemoglobin in THYB_NTA_ experience iron depletion compared with those growing in THYB_CHX_

**Fig 7:**
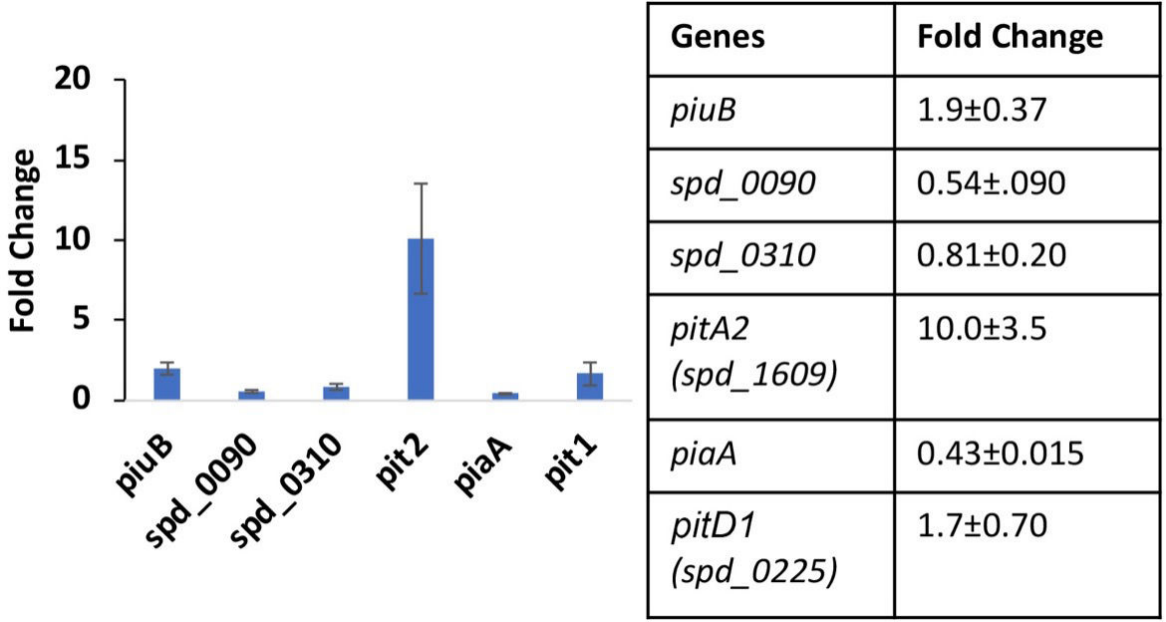
The expression of iron uptake genes is induced in cells grown using met-hemoglobin as the source of iron in THYB_NTA_ compared with cells cultivated in THYB_CHX_. Cells grown in THYB_CHX_ or THYB_NTA_ supplemented with 5-µM hemoglobin were harvested at the mid-log phase (OD_600_ ~0.4), RNA was prepared, and gene expression was determined by qRT-PCR. Data derived from two bioreplicates processed each in duplicates are shown in a bar graph and a table format. Values smaller than one indicate downregulation.

We noticed that all three strains tested for growth on met-hemoglobin iron grew faster and reached higher biomass in THYB_CHX_ repleted with met-hemoglobin compared to repleted THYB_NTA_ (Fig. 3C and D compared with Fig. 3E and F). To gain more insights into pneumococcal physiology during cultivation using met-hemoglobin as a single source of iron, we compared the expression of the pneumococcal genes implicated in iron or heme uptake between cells grown in THYB_CHX_ or THB_NTA_ supplemented with limiting amounts (5 µM) met-hemoglobin. RNA was prepared from samples collected at the mid-logarithmic phase of growth, and the expression of selected iron homeostasis genes was analyzed by RT-PCR (Fig. 7). Although both media were supplemented with equal amounts of met-hemoglobin, we observed differential expression of several transporters. The most pronounced change was the 10-fold induction of *pitA2* (*spd_1609*) during growth in THYB_NTA_ compared with the Chelex-treated THYB. Smaller but more significant induction of the *piuB* (1.9-fold) and *pitD1* (spd-0225, 1.7-fold) was also observed. The elevated expression of *pitD1* during growth in THYB_NTA_ compared with THYB_CHX_ is consistent with the observation that the ∆*piuBCDA* strain imported less heme than the WT during growth in this medium, while the *piu* loss did not impact heme content during growth in THYB_Chx_. Interestingly, cells grown on hemoglobin iron in the presence of NTA downregulate the expression of *spd_0090* (0.54-fold)*,* which is part of a recently described heme importer, and of *piaA* (0.43-fold), a ferrochrome-binding protein (18). The repression of *spd_0090* and *piaA* genes suggests different regulation modalities for these transporters.

## DISCUSSION

**Fig 8:**
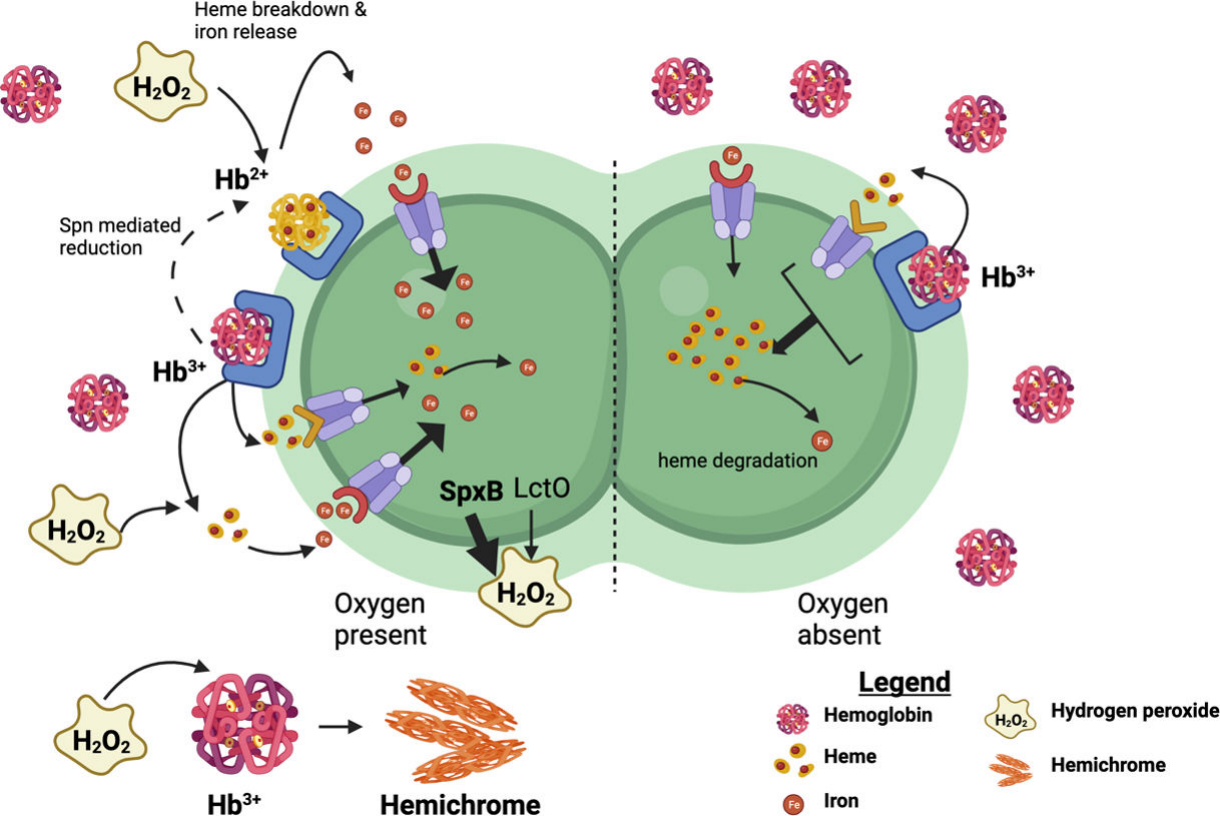
A proposed model for pneumococcal growth on met-hemoglobin as an iron source. The left side represents aerobic environments where the SpxB and LctO are producing H_2_O_2_ that escapes to the extracellular milieu. We propose that met-hemoglobin (Hb^3+^) is captured on the surface and is either reduced (to Hb^+2^) by surface reductases or heme is released by receptor-mediated processes. Subsequently, reduced hemoglobin and liberated heme are vulnerable to H_2_O_2_ attack resulting in the release of iron that is imported by iron-dedicated transporters. Extracellular met-hemoglobin cells form hemogloblin precipitants (hemichrome) following reactions with H_2_O_2_. The right side represents anoxic environments where pneumococcus does not produce H_2_O_2_. Here, pneumococcus binds met-hemoglobin and imports heme as a primary iron source via heme transporters. Image was generated by Biorender.com.

The human pathogen, *S. pneumoniae*, requires iron and can readily obtain the metal from heme and host hemoproteins, with hemoglobin the most growth-beneficial source compared with other iron sources (9). Still, the molecular mechanisms by which pneumococci obtain and import heme are only partially described, and the literature contains inconsistent reports regarding the function of transporters implicated in iron acquisition (15 – 17, 36). Additionally, it is unknown how this important pathogen degrades heme to liberate iron. One confounding factor that was largely overlooked in previous studies of iron metabolism in *S. pneumoniae* is that in the presence of oxygen, the bacterium produces and releases to the extracellular environment copious amounts of H_2_O_2_, which can rapidly interact with free and hemoglobin-bound iron. While H_2_O_2_ degrades both ferrous heme and ferric heme that are free in solution, the fate of the heme that is bound to hemoglobin depends on the iron redox state (20 – 23, 31, 32). In this study, we began describing the role of H_2_O_2_ production in iron acquisition by *S. pneumoniae*, uncovering intimate relationships between iron metabolism and H_2_O_2_ production as illustrated in Fig. 8.

Examining the impact of met-hemoglobin on pneumococcal cultures showed that the millimolar concentration of H_2_O_2_ produced by the pneumococcal SpxB and LctO enzymes is removed from the medium in the presence of micromolar amount of met-hemoglobin (Fig. 1B). This catalytic removal of H_2_O_2_ is consistent with the catalase-like activity of met-hemoglobin, whereby H_2_O_2_ acts as both an electron donor and acceptor in an oxidation-reduction cycle of Hb-Fe^+3^ and Hb-Fe^+4^ (32). H_2_O_2_ removal likely reduces the consequential oxidative stress pneumococcus experiences during growth likely contributing to the dramatic growth benefits we observed when *S. pneumoniae* is cultivated in the presence of met-hemoglobin (9). Interestingly, *spxB* expression augments the release of Ply by *S. pneumoniae* (29), which in turn promotes erythrocyte lysis and the release of hemoglobin to the medium (29). Hence, it is plausible that *in vivo*, H_2_O_2_-producing pneumococci (such as those colonizing the respiratory tract) also benefit from the removal of H_2_O_2_ in their vicinity by met-hemoglobin.

Met-hemoglobin is stable in a cell-free medium, but it is falling out from the medium during incubation with growing streptococci. We notice a faster decrease in soluble met-hemoglobin level in the WT cultures compared with the ∆*spx*B∆*lctO* mutant (Fig. 1C). Oxidation of met-hemoglobin by H_2_O_2_ produces reactive amino acid radicals, destabilizes the globin chain, and promotes met-hemoglobin conversion to hemichrome (met-hemoglobin complexes in which a histidine residue, distal or external, binds to the iron sixth position). Since hemichrome readily precipitates out of solution forming Heinz bodies *in vivo* (37), it is likely that hemichrome formation in the presence of H_2_O_2_ enhances the loss of met-hemoglobin from the solution. We observed met-hemoglobin precipitation also in ∆*spxB*∆*lctO* cultures although less than with the WT. Hence, additional streptococcal-dependent but H_2_O_2_-independent mechanisms, possibly the reduction in the medium pH during bacterial growth, also lead to met-hemoglobin denaturation (38). The hemoglobin that falls out of the solution likely supports the robust biofilm growth we observe when *S. pneumoniae* is grown in the presence of met-hemoglobin (10).

It is well documented that H_2_O_2_ degrades free heme and heme that is bound to oxyhemoglobin (20, 22, 23, 31), while its reactions with met-hemoglobin heme do not lead to iron release (23). Therefore, we were surprised to find that incubation of met-hemoglobin with H_2_O_2_-producing streptococci led to accumulation of free iron in the medium, albeit in low amounts (Fig. 2A). Extracellular iron accumulation was not observed with the ∆*spx*B∆*lctO* mutant, and externally added catalase blocked this buildup in the WT cultures (Fig. 2A). These observations suggest that iron is released extracellularly from met-hemoglobin heme by a H_2_O_2_-dependent process. Incubation of met-hemoglobin in a spent medium that contains H_2_O_2_ did not lead to iron release from met-hemoglobin (Fig. 2B), indicating that extracellular degradation of the heme in met-hemoglobin also requires the presence of pneumococcal cells.

Pneumococcus has two surface-exposed thioredoxin proteins, Etrx1 and Etrx2, that help them cope with oxidated stress by maintaining a reductive outward environment (32). It seems possible that these enzymes reduce the ferric iron in met-hemoglobin molecules, making it suspectable to degradation by H_2_O_2_. Alternatively, binding of met-hemoglobin to pneumococcal receptors may prompt local heme release, which is then degraded by H_2_O_2_. The contribution of H_2_O_2_-mediated degradation of free heme that was pumped out by pneumococci cannot be overruled as well. Still, the low amounts of iron found in the medium (Fig. 2A) compared with the relatively high concentration of met-hemoglobin present (Fig. 5) support a model by which there is a localized iron release from met-hemoglobin, which is in turn taken up by the cells.

To examine how extracellular heme degradation impacts pneumococcal iron metabolism, we compared growth and cellular heme and iron content among pneumococci grown on met-hemoglobin iron. All tested strains were able to grow (Fig. 3) and accumulated heme (Fig. 4A and B) in either THYB_CHX_ or THYB_NTA_ supplemented with met-hemoglobin. The *piuBCDA* mutant accrued significantly lower heme amounts compared with the parent WT strain when grown in THYB_NTA_ (orange and blue symbols, Fig. 4B). This phenotype indicates that active uptake promotes cellular heme accumulation and establishes the Piu proteins as an important mechanism when *S. pneumoniae* is dependent on external heme supply. Despite these differences in heme content, the growth of the ∆*piuBCDA* mutant was like that of the WT parent (Fig. 3), consistent with the presence of additional transporters whose activity compensates for the loss of the Piu proteins.

Cellular heme levels peaked during the mid-logarithmic phase of growth in most cultures and then decreased. We suggest that intracellular heme catabolism and possibly heme export facilitated this decrease in heme levels during long incubation. Since the free iron in the medium is chelated, pneumococci cultivated in THYB_NTA_ with met-hemoglobin must degrade the heme in the intracellular compartment to obtain the metal. Hence, the growth of the *∆spxB∆lctO* on met-hemoglobin in THYB_NTA_ indicates the presence of pneumococcal mechanisms for intracellular heme degradation that are independent of H_2_O_2_. Interestingly, the only sample in which the heme level did not decline after prolonged incubation was that of *∆spxB∆lctO* cells grown on met-hemoglobin in THYB_CHX_. This observation suggests that H_2_O_2_ may also contribute to intracellular heme degradation. It is also possible that H_2_O_2_-independent heme degradation comes into play only in the absence of H_2_O_2_ production and without a free iron source (such as in THYB_NTA_). The pneumococcal mediators of catalytic heme degradation are unknown at this time*,* as heme-degrading enzymes were not described, and the pathogen does not encode homologs of recognized heme-degrading proteins (39). Nevertheless*,* catalytic heme degradation is likely critical during infection in sites with low oxygen tension (where pneumococci do not produce H_2_O_2_).

We found less heme in the WT compared with the ∆*spxB*∆*lctO* mutant when the bacteria were grown on met-hemoglobin iron (blue and gray symbols, Fig. 4A and B). These differences in heme content were bigger when the cells were cultivated in THYB_CHX_ than in THYB_NTA_ (5-fold versus 2.5-fold, respectively) in which pneumococcal growth was dependent on heme import. Despite the difference in cellular heme, both strains contained equal amounts of iron (Fig. 4C). These findings suggest that the WT strain incorporates mostly free iron under our experimental growth conditions. Intracellular heme degradation or export may be enhanced in the H_2_O_2_-producing cells. Still, the reduction in heme content (about a quarter) within the WT strain when grown in THYB_CHX_ compared with THYB_NTA_ (blue symbols, Fig. 4A and B) supports the notion that the WT strain takes up free iron although it is cultivated in THYB_CHX_ with met-hemoglobin as the only source of iron supporting extracellular heme degradation.

We found that inactivation of the genes for the H_2_O_2_-producing enzymes, *spxB* and *lctO*, resulted in wide activation of transporters for iron (*pitD1* and *pitA2*, 8.7–9-fold), heme (*spd_0090*, 5.2-fold), and an unknown iron complex (*piaA*, 1.6-fold; Fig. 6). We hypothesize that the likely increase in iron import contributes to the resistance to iron stress exhibited by the ∆*spxB*∆*lctO* mutant (Fig. 4A and B). The strong repression of the *piuB* expression (0.067) is intriguing and may reflect the complexity of *piu* regulation. The transcription of the *piu* genes is regulated by multiple proteins including CodY and the orphan response regulator RitR (40, 41) and environmental conditions such as oxygen tension, oxidation stress, and nutrition status. Nevertheless, the significant activation of several iron-related transporters in the ∆*spxB*∆*lctO* strain strongly associates H_2_O_2_ production and iron metabolism in *S. pneumoniae*.

All pneumococcal strains grew to higher cell density on met-hemoglobin iron in THYB_CHX_ compared with THYB_NTA_ (Fig. 3C–F). In addition, WT pneumococci grown on 5-µM met-hemoglobin as an iron source exhibited 10-fold activation of the metal iron transporter, *pitA2*, and a small increase in the expression of *pitD1* and *piuB* genes (1.7- and 1.9-fold respectively) when grown in THYB_NTA_ compared with THYB_CHX_ (Fig. 7). This observation indicates that the transcription of the *pitD1*, *pitA2*, and *piuB* genes is responsive to iron (whose availability in the medium is limited in THYB_NTA_) and not heme. The molecular mechanism for such iron regulation remains elusive; however, since *S. pneumoniae* does not code for obvious homologs of the iron-dependent repressors from the Fur families, a potential DtxR family member named SmrB has been identified. SmrB resembles the regulator of Mn uptake in *Treponema pallidum*, TroR, but its role in iron metabolism is unknown (42). Both media were supplemented with limiting but equal amounts of met-hemoglobin. Hence, it seems that pneumococci that are not allowed to benefit from external heme degradation by H_2_O_2_ perceive more severe iron stress compared with those that grow without an external chelator. Together, these observations provide additional support to the idea that extracellular heme degradation by H_2_O_2_ provides pneumococci with nutritional iron from met-hemoglobin.

*S. pneumoniae* is a versatile pathogen that can spread from the upper respiratory tract and survive in multiple niches in the host (2). In this study, we establish that H_2_O_2_ is a key mediator in pneumococcal use of the host heme and hemoproteins as iron sources. We expect that *in vivo*, the H_2_O_2_-dependent pathway is important particularly during nasopharyngeal and lung colonization, while during invasive progressions, such as with bacteremia, pneumococci likely rely on additional mechanisms for iron acquisition that are independent of H_2_O_2_. Sickle cell anemia (SCA) is another *in vivo* condition where the interactions between pneumococcal-produced H_2_O_2_ and hemoglobin described in this study may contribute to the infection outcome. Patients with SCA suffer from invasive pneumococcal disease at a 100 times higher rate than non-SCA patients despite not having distinguishable differences in their immune system (43). Interestingly, SCA hemoglobin (HbS) is more sensitive to H_2_O_2,_ with the ferrous form (HbS-Fe^2+^) auto-oxidizing to the ferric (HbS-Fe^3+^ or met-HbS) at a nearly double the rate of normal hemoglobin (43). It is, therefore, likely that endogenously produced H_2_O_2_ enhances iron release, increases hemichrome generation, and promotes pathology in these patients.

## MATERIALS AND METHODS

### Bacterial growth and media

Frozen stocks of *S. pneumoniae* were preserved in skim milk-tryptone-glucose-glycerin (STGG) as described (44) and stored at −80°C. *S. pneumoniae* cells from STGG stock were plated on tryptic soy blood agar plates (BAPs) (Becton Dickinson) and incubated at 37°C. Cells collected from BAPs following overnight incubation were used to inoculate fresh medium in a starting OD_600_ of 0.05. Pneumococci were grown in THYB containing 0.5% (wt/vol) yeast extract (Becton Dickinson), iron-depleted THYB (THYB_CHX_ or THYB_NTA_), or iron-depleted THYB supplemented with hemoglobin. THYB_CHX_ was prepared by adding 5% (wt/vol) Chelex-100 resin (Sigma-Aldrich) to THYB and incubating with continued mixing overnight. The resin was removed using a 0.45-micron filter, and the medium was supplemented with 2-mM MgCl_2_ and 100-µM CaCl_2_. THYB_NTA_ was prepared by adding 3-mM NTA (Sigma-Aldrich), 0.55-mM ZnSO_4_, and 0.55-mM MnCl_2_ to fresh THYB followed by filter sterilization (0.45-micron filters). The metal composition of the chelated media was determined experimentally by supplementing back with different transition metals and testing growth; we chose eventually to include only the metals whose absence negatively impacted growth. We cannot exclude the possibility that a reduction in non-growth essential metals might have impacted the finding. Hemoglobin stock solutions were prepared fresh by resuspending the lyophilized power of human hemoglobin (Sigma-Aldrich) in 1× PBS (1 mM). Hemoglobin was then added at the indicated concentration, and the medium was then filter sterilized (0.45-micron filters). Pneumococci were grown in 96-well flat bottom tissue culture plates at 37°C in a Multiscan SkyHigh Spectrophotometer (Thermo Scientific). Optical density was measured hourly after brief shaking.

### Hemoglobin spectroscopic measurements

Absorbance spectra (250–700 nm) of hemoglobin samples in PBS or THYB were determined using a DU730 LifeScience UV/VIS or Beckman-Coulter DU 730 UV/Vis spectrophotometer in a quartz cell with an optical path length of 10 mm. In some experiments, 10-µM hemoglobin was incubated with 10-mM DTT (Sigma-Aldrich) or 30-µM potassium ferricyanide (Thermo Fisher) for 1 hour in PBS prior to spectroscopic measurements.

### Bacterial strains and mutant construction

The bacterial strains and plasmids used in the study are listed in Table 1 and the primers in Table 2. The *∆spxB∆lctO* knockout and its isogenic Ω*spxB-lctO* complemented strain were engineered *S. pneumoniae* D39 strain as described for the construction of this mutation and complementation in TIGR4 strain (45). The ∆*piuBCDA* mutant was created by replacing the *piuBCDA* gene cluster with the *ermC* gene from plasmid pJRS233 (46). A chimeric fragment consisting of the *ermC* gene, expressed from its native promoter, preceding with a 1,013-bp fragment of D39 *piuB* upstream region (including the first 213 bp of *piuB* open reading frame [ORF]) and proceeding with an 818-bp fragment with *piuA* downstream area (including the last 534 bp of *piuA* ORF), was cloned into pAF103 by Gibson cloning using the GeneArt Seamless Cloning Kit (Invitrogen) and transformed into One Shot TOP10 *Escherichia coli* strain. A linear fragment (1,252 bp) containing *ermC* flanked with *S. pneumoniae* chromosomal fragments was amplified from pAF103 and introduced into competent D39 WT cells. Pneumococcal clones harboring the ∆*piuBCDA* mutation were selected on erythromycin (0.5 µg/mL), and the chromosomal deletion of *piuABCD* genes was confirmed by PCR analysis.

**TABLE 1:** Strains and plasmids

Strain or plasmid	Description	Source or reference
*S. pneumoniae* strains		
D39	Avery strain, clinical isolate, WT (capsular serotype 2), CSP1	Lanie et al. (47)
D39 *∆spxB∆lctO*	D39 derivative with double knockout of *spxB* and *lctO* genes	This study
D39 Ω*spxB-lctO*	D39 *∆spxB∆lctO* mutant expressing the *spxB* and *lctO* genes from a heterologous chromosomal location	This study
D39 *∆piuBCDA*	D39 derivative with ∆*piuBCDA*::*ermC* mutation (Erm^R^)	This study
*E. coli* strain		
One Shot TOP10	Cloning strain	Invitrogen
Plasmid		
pAF103	Plasmid for allelic replacement of *∆piuBCDE::ermC* with pUC19 backbone, Amp^r^	This study

**TABLE 2:** Primers used in this study

Amplified region	Primer name	Sequence (5′−3′)	Comments
pUC19 linear vector (Invitrogen)	ZE 730	GTCTGGAAGGCATGCAAGCTTGGCGTAATCAT	Sense primer for cloning of the pUC19 segment of pAF103
	ZE 731	GAACATGAGTACCGAGCTCGAATTCACTGGCC	Antisense primer for cloning of the pUC19 segment of pAF103
5′ piuB and upstream region (D39)	ZE 732	CTCGGTACTCATGTTCTCTTCGACTGCTTCTC	Sense primer for cloning of the left arm of *piu* segment of pAF103
	ZE 733	CACACGGTGGTAATAGTCTGCATGAGAAGGCC	Antisense primer for cloning of the left arm *piu* segment of pAF103
*ermC* (pJRS233)	ZE 734	CTATTACCACCGTGTGCTCTACGACCAAAACT	Sense primer for cloning of the *ermC* segment of pAF103
	ZE 735	TTGCTTGCCAAAGCTGCCGACAACACGGGAGC	Antisense primer for cloning of the *ermC* segment of pAF103
3′ *piuA* and downstream region (D39)	ZE 736	CAGCTTTGGCAAGCAAGGATGACTACTGGACT	Sense primer for cloning of the right arm of *piu* segment of pAF103
	ZE 737	TGCATGCCTTCCAGACCACTTTTCCCTTAAAC	Antisense primer for cloning of the right arm *piu* segment of pAF103
*gyrB*	ZE 661	GGCACTGTATGGTATCACACAAG	qPCR
	ZE 662	TCTCTAAATTGGGAGCGAATGTC	
*piuB*	ZE 864	TGATTTCGACCAGCAGACCTG	qPCR
	ZE 865	CTGTACTCGGTGCAGCAAACTG	
*pitA2* (*spd_1609*)	ZE 149	ATTGCCCGTCCTGTACCACC	qPCR
	ZE 150	TGCTGCTTGCTCTGGAGGTT	
*piaA*	ZE 151	AAAAATGGTGCCGTTGCTGT	qPCR
	ZE 152	AAAGTGGTGTTGGAGTGCATGA	
*spd_0225* (*pitD1*)	ZE 153	CCAAGCGAAGTTTGGCCTCC	qPCR
	ZE 154	CACCTTGGCATGCGCCTTAG	
*spd_0310*	ZE 155	TGATCAAGGCAGCGGCTGTA	qPCR
	ZE 156	GAAACTGGTGGACCAGCCCT	
*spd_0090*	ZE 171	ACGGTCCAGAAGGCAAGAACT	qPCR
	ZE 172	ACCAGTGTTCCATCCACCCA	

### Quantification of H_2_O_2_ concentration in pneumococcal growth media

Pneumococcal cells collected from BAP following overnight growth at 37°C were used to inoculate fresh THYB or THYB supplemented with 20-µM met-hemoglobin at OD_600_ 0.05 (6 mL in 15-mL falcon tubes). The supernatant was prepared from culture samples by centrifugation and filtration (0.45 micron), and the hemoglobin was removed by filtration with Ultra-15 centricon filters (molecular weight cutoff of 30,000, Amicon). The sample H_2_O_2_ content was measured using the Quantitative Peroxide Assay Kit (Thermo Fisher) per the manufacturer’s instructions. A serial dilution of 30% H_2_O_2_ in THYB was used to generate a standard curve, from which we derived the H_2_O_2_ concentration in media samples.

### Measurement of free iron concentration in pneumococcal growth media

We determined the concentration of free iron in the medium samples using an optimized ferrozine (3-(2-pyridyl)-5,6-diphenyl-1,2,4-triazine-p,p′-disulfonic acid monosodium salt hydrate, Milli Sigma) based assay as described (33). Briefly, hemoglobin was incubated in 2-mL THYB or in spent THYB (collected from overnight pneumococcal cultures) in 12-well flat bottom microtiter plates, at 37°C. Hemoglobin-free samples collected at different time points were treated with 50-mM ascorbic acid and incubated with the 50-mg ferrozine/mL and 500-mM potassium acetate (pH 5.5) in 96-well flat bottom plates for 2.5 hours prior to reading. Absorbance at 562 nm was determined after 135 minutes of incubation at 37°C, and iron concentration in the medium was calculated using a standard curve. The same was done with the cell- and hemoglobin-free supernatant samples collected from pneumococcal cultures grown in THYB with hemoglobin with or without 200-U/µL catalase (Sigma-Aldrich). Net iron released into the culture during growth was calculated by subtracting the iron values determined in un-inoculated THYB containing hemoglobin.

### Determination of heme content

Fresh THYB_CHX_ or THYB_NTA_ was supplemented with 20-µM hemoglobin, inoculated with *S. pneumoniae* OD_600_ 0.05 in 12-well flat bottom microtiter plates and incubated at 37°C. Six-milliliter culture samples (standardized to OD_600_ 1.0) were collected at different time points. The cells were harvested by centrifugation, washed three times with PBS, resuspended in 2-mL DMSO, and sonicated (20% amplitude for 30 seconds). Heme was extracted by acidified chloroform, and its concentration was determined using a standard curve made with hemin solutions in DMSO as described (34). Briefly, 2 mL of 50-mM glycine buffer, pH 2.0, 0.1 mL of 4 N HCl (pH 2.0), 0.2 mL of 5-M NaCl (pH 2.0), and 2 mL of chloroform:isopropanol were added to the cell lysates. The reactions were mixed vigorously and were allowed to incubate at room temperature for 1 minute. The absorbance of the organic phase at 388, 450, and 330 nm was recorded and fed into the correction equation Ac  =  2  ×  A388 − (A450 + A330). Hemoglobin (if it was present in the culture medium) was removed by filtration before heme extractions.

### Total iron by ICP-MS

Fresh THYB_CHX_ or THYB_NTA_ supplemented with 20-µM hemoglobin was inoculated with *S. pneumoniae* grown on BAPs (starting OD_600_ 0.05) and allowed the culture to grow in 12-well microtiter plates at 37°C for 6 hours. Six-milliliter culture samples (standardized to OD_600_ 1) were harvested, washed three times with phosphate-buffered saline, and sent for ICP analysis (Center for Applied Isotope Studies, University of Georgia, Athens, GA) as described (9).

### qRT-PCR analysis

Quantitative RT-PCR (qRT-PCR) analysis was carried out using the Power SYBR Green RNA-to-Ct 1-Step Kit (Applied Biosystems) and StepOne DNA PCR machine (Applied Biosystems) according to the manufacturer’s specifications. A 25-ng RNA was used per qRT-PCR reaction, and each reaction was done in duplicates. Primers used for qRT-PCR are listed in Table 2. The relative expression was normalized to the endogenous control *gyrB* gene, and fold changes were calculated using the comparative 2^−ΔΔCT^ method.
